# The constitutive activity of the virally encoded chemokine receptor US28 accelerates glioblastoma growth

**DOI:** 10.1038/s41388-018-0255-7

**Published:** 2018-04-30

**Authors:** Raimond Heukers, Tian Shu Fan, Raymond H. de Wit, Jeffrey R. van Senten, Timo W. M. De Groof, Maarten P. Bebelman, Tonny Lagerweij, Joao Vieira, Sabrina M. de Munnik, Laura Smits-de Vries, Jody van Offenbeek, Afsar Rahbar, Diane van Hoorick, Cecilia Söderberg-Naucler, Thomas Würdinger, Rob Leurs, Marco Siderius, Henry F. Vischer, Martine J. Smit

**Affiliations:** 1 Amsterdam Institute for Molecules, Medicines and Systems (AIMMS), Division of Medicinal Chemistry, Faculty of Sciences, Vrije Universiteit, De Boelelaan 1108, Amsterdam, 1081 HZ The Netherlands; 2 Neuro-oncology Research Group, Cancer Center Amsterdam, VU University Medical Center, De Boelelaan 1117, Amsterdam, 1081 HV The Netherlands; 3 Ablynx N.V., Technologiepark 21, Zwijnaarde, 9052 Belgium; 4 Department of Medicine Solna, Experimental Cardiovascular Research Unit and Department of Medicine and Neurology, Center for Molecular Medicine, Karolinska Institute, Stockholm, 171 77 Sweden

## Abstract

Glioblastoma (GBM) is the most aggressive and an incurable type of brain cancer. Human cytomegalovirus (HCMV) DNA and encoded proteins, including the chemokine receptor US28, have been detected in GBM tumors. US28 displays constitutive activity and is able to bind several human chemokines, leading to the activation of various proliferative and inflammatory signaling pathways. Here we show that HCMV, through the expression of US28, significantly enhanced the growth of 3D spheroids of U251− and neurospheres of primary glioblastoma cells. Moreover, US28 expression accelerated the growth of glioblastoma cells in an orthotopic intracranial GBM-model in mice. We developed highly potent and selective US28-targeting nanobodies, which bind to the extracellular domain of US28 and detect US28 in GBM tissue. The nanobodies inhibited chemokine binding and reduced the constitutive US28-mediated signaling with nanomolar potencies and significantly impaired HCMV/US28-mediated tumor growth in vitro and in vivo. This study emphasizes the oncomodulatory role of HCMV-encoded US28 and provides a potential therapeutic approach for HCMV-positive tumors using the nanobody technology.

## Introduction

Glioblastoma (GBM) is the most common type of human brain cancer and is characterized by severe aggressiveness. The disease accounts for over 45% of all malignant primary brain tumors with an age-adjusted incidence rate of 3 cases per 100,000 person-years [1]. Despite aggressive therapeutic intervention by chemo/radiotherapy and surgical resection [2], the clinical outcome is extremely poor with a median survival of only 15 months and overall 5-year survival rate of <5% [1]. Human cytomegalovirus (HCMV) is widely spread in the population and establishes life-long latency in immunocompetent individuals [3, 4]. Upon immunodeficiency (e.g., AIDS patients, allograft recipients, tumor-associated inflammation), the virus can be reactivated and cause severe pathologies [5]. HCMV DNA and proteins have been detected in most GBM samples (50–90%) as well as in several other cancer types, with surrounding healthy tissues being HCMV negative [6, 7]. The detection of HCMV mRNA and/or protein in GBM tissues is hampered by their restricted expression [8, 9]. Nevertheless, treatment of HCMV-positive xenograft tumors in mice models with antiviral compounds reduces tumor growth and HCMV-directed immunotherapy show a promise to improve patient outcome [10–13]. Moreover, moderate-to-high-grade HCMV infection was associated with poor survival of GBM patients [14], which was extended by long-term adjuvant therapy with the HCMV-inhibitor valganciclovir [15, 16]. While there is consensus on the potential oncomodulatory role of HCMV in GBM [17], the mechanism by which HCMV exerts these effects remain incompletely understood.

HCMV encodes four viral G protein-coupled receptors (viral GPCRs: US27, UL78, UL33 and US28). Of these, US28 is the best characterized and shares homology to the human chemokine receptor family. US28 is located on the virion and is subsequently expressed in HCMV-infected cells during all stages (i.e., latent and active states) of the viral life cycle [18]. Detection of US28 DNA, RNA or protein in GBM tissues requires sensitive techniques and particular immune-histochemical epitope unmasking approaches [8]. Under these conditions, US28 was detected in 53–65% of the 35 different GBM tissues tested [19]. US28 displays oncomodulatory activity via constitutive and promiscuous G protein coupling and subsequent signaling towards a broad panel of transcription factors and cytokines (e.g., NF-κB, STAT3, IL-6, TCF/LEF and HIF-1) that are involved in cell proliferation, survival, migration, angiogenesis and inflammation [19–25]. Additionally, US28 binds and internalizes a broad spectrum of human chemokines (e.g., CCL2/5, CX3CL1), which might contribute to immune evasion of infected host cells [3]. US28 has also been shown to be oncogenic, leading to tumor development when expressed in mouse 3T3 cells [22]. Moreover, the detection of US28 in GBM samples, together with its constitutive activity and promiscuous oncomodulatory signaling, turn US28 into a potential novel target in GBM [19–22].

In recent years, the development of antibody-based biologicals to target chemokine receptors is on the rise [26]. In particular, small antibody fragments derived from heavy-chain-only antibodies, also referred to as variable domains of heavy-chain-only antibodies (VHHs) or Nanobodies® (Nbs) have shown great potential as detection tools, crystallization-chaperones and/or therapeutics for several receptor classes, including GPCRs [26–28]. Recently, nanobodies were developed to successfully target and inhibit the human chemokine receptors CXCR2, CXCR4 or CXCR7 [29–31]. Bivalent nanobodies were shown to display inverse agonistic activities against a constitutively active CXCR4 mutant and CXCR2 [29, 30]. Furthermore, an intracellular-surface binding nanobody was generated to obtain high resolution (2.9 Å) crystal structures of the active conformation of US28 [27].

In this study, we examined the consequence of US28 expression in the context of GBM in more detail and evaluated US28 as a therapeutic target for HCMV-associated GBM. We generated nanobodies that specifically inhibited ligand-dependent and constitutive US28 activity, which consequently impaired US28-dependent GBM growth in vitro and in vivo in an orthotopic xenograft model in mice. Our findings suggest an important role for US28 signaling in modulating HCMV-associated GBM growth. The US28-specific nanobodies are important research and diagnostic tools, which may further substantiate a role for HCMV-encoded US28 in HCMV-associated GBM. Moreover, they have therapeutic potential to improve the clinical outcome for patients with this devastating disease.

## Results

### HCMV-encoded chemokine receptor US28 enhances GBM growth

**Fig. 1:**
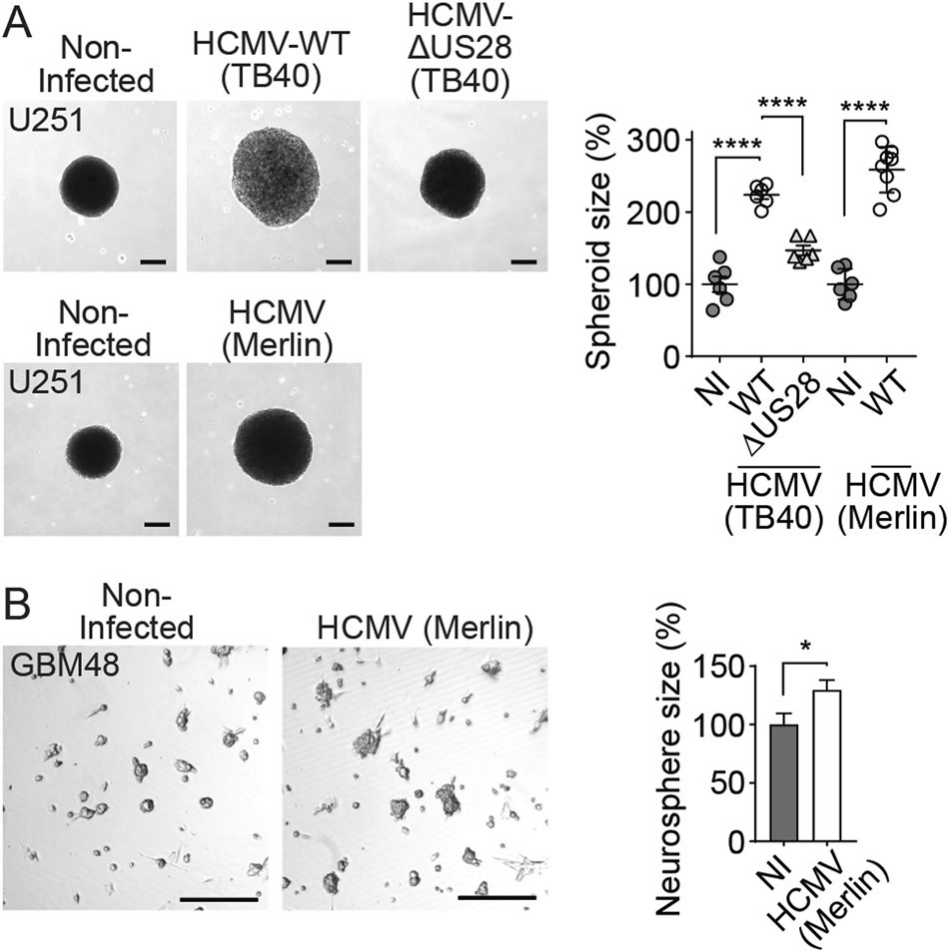
US28 mediates HCMV-enhanced GBM growth. **a** Representative spheroids and spheroid size of uninfected U251 cells, or U251 cells infected with HCMV strain TB40/E wild type, HCMV in which US28 was deleted (HCMV-∆US28) or strain Merlin wild type. Spheroids were generated using the hanging droplet method followed by culture on 0.75% agarose (*n* = 6 spheroids per group). **b** Neurospheres of GBM48 primary GBM cells upon infection with HCMV strain Merlin (*n* = > 80 neurospheres per group). Representative plots are shown. All scale bars represent 250 μm. Sizes of individual spheroids or neurospheres are plotted as percentages of non-infected with mean ± SEM. *P < 0.05, ****P < 0.0001 (unpaired *t* test)

**Fig. 2:**
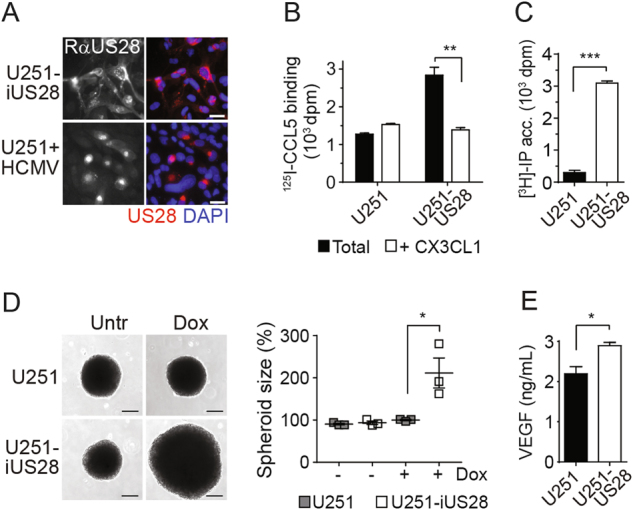
US28 signaling in enhances growth of GBM spheroids. **a** Inducible expression of US28 in U251-iUS28 cells. Expression of US28 was induced with doxycycline. Bottom panel shows US28 expression in HCMV Merlin-infected U251 cells. US28 was stained in fixed/permeabilized cells using polyclonal rabbit-anti-US28 antibodies directed against the C-terminus of US28. Scale bars represent 20 μm. **b** Whole-cell binding of ^125^I-CCL5 to U251-iUS28 cells shows membrane expression of US28 upon induction with doxycycline. Specific displacement of ^125^I-CCL5 was performed with an excess of CX3CL1. **c** Constitutive US28-mediated PLC activation U251-iUS28 cells upon induction of US28 expression as subsequently determined by inositol phosphate ([^3^H]-IP) accumulation. **d** US28 expression in U251-iUS28 cells induces growth of GBM spheroids. Spheroids were generated by the hanging droplet method. A representative graph of spheroid size quantification is shown. Individual spheroid sizes are plotted as percentages with mean ± SEM (*n* = 6 spheroids per group). **e** Spheroids from U251-iUS28 cells secrete VEGF upon induction of US28 expression. The data show mean ± SEM. *P < 0.05, **P < 0.01, ***P < 0.001 (unpaired *t* test)

Infection of U251 MG GBM cells with HCMV (strain TB40/E) significantly enhanced the size of 3D spheroids (Fig. 1a). This HCMV-induced growth was significantly reduced when these cells were infected with a HCMV variant in which US28 was deleted (HCMV-∆US28), despite comparable infection rates (Supplementary Fig. 1). This observation suggests a key role for US28 in the oncomodulatory properties of HCMV in these GBM cells. Infection of these cells with the clinically more relevant HCMV strain Merlin resulted in a significant enhancement of U251 spheroid size (Fig. 1a) and neurospheres of GBM48 primary GBM cells (Fig. 1b). Both U251 and GBM48 cells only expressed US28 upon HCMV-infection, as determined by qPCR and immunofluorescence microscopy (Supplementary Fig. 2). To study the role of US28 in GBM tumor development in vitro and in vivo in more detail, we established a doxycycline-inducible US28 GBM cell line (U251–iUS28) and developed an orthotopic intracranial GBM model in mice using this cell line. After induction of gene expression, US28 protein levels were confirmed using fluorescence microscopy and specific ^125^I-CCL5 binding (Fig. 2a, b). In line with previous studies, doxycycline-induced US28 expression resulted in a significantly enhanced intracellular accumulation of inositol phosphates (IP) (Fig. 2c) [21]. Moreover, US28 expression significantly enlarged U251 spheroids (Fig. 2d), accompanied with an increase in VEGF secretion (Fig. 2e). These data confirms that US28 exhibits oncomodulatory signaling properties in GBM cells in vitro [25].

**Fig. 3:**
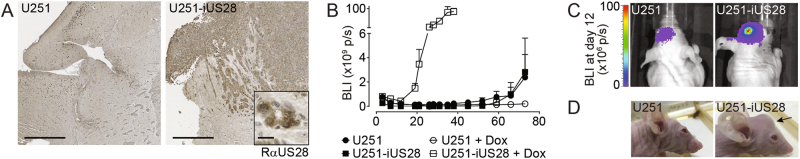
Constitutive activity of US28 enhances GBM growth. **a** Sections of paraffin-embedded brains from US28-expressing orthotopic GBM model in mice, stained with hematoxylin (blue) and rabbit-anti-US28 antibodies (brown). Scale bars represent 100 μm (large panels) or 30 μm (small panel). **b**–**d** Orthotopic GBM model generated with inducible U251-FM-iUS28 cells or parental U251-FM cells in striatum of mice treated with either sucrose alone or doxycycline/sucrose in their drinking water. **b**, **c** In vivo tumor growth as quantified by bioluminescence imaging (*n* = 6 mice per group). **d** US28-positive tumors were externally visible (arrow)

In order to study this phenotype in vivo, U251 and U251-iUS28 lines constitutively expressing firefly luciferase/mCherry (FM) were generated to allow in vivo tumor size quantification via bioluminescent imaging (BLI) [32]. These U251-FM-iUS28 cells showed similar increases in IP signaling (Supplementary Fig. 3) and were subsequently used in an orthotopic GBM model in the striatum of mice brains (Fig. 3a–d). The inducible expression of US28 in these brain tumors was confirmed by immunohistochemistry on paraffin-embedded brain tissue sections (Fig. 3a). While control and non-induced tumor expansion initiated ~40 days after surgery, growth of US28-expressing tumors was already evident after 10 days and showed a significantly accelerated rate of tumor growth (Fig. 3b). In contrast, U251-FM cells did not form tumors in mice upon doxycycline treatment. The US28-enhanced growth was also apparent by a clear invasive and extracranial tumor growth (Fig. 3c, d), which are obvious signs of a more aggressive tumor phenotype. These data indicate that US28 expression accelerates GBM tumor growth.

### Generation of US28-targeting nanobodies

**Fig. 4:**
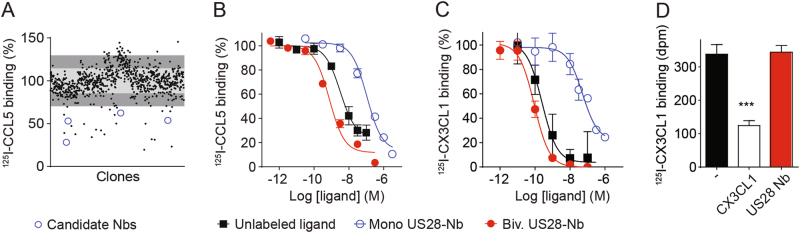
US28-targeting nanobodies displace chemokine binding. **a** Displacement of ^125^I-CCL5 from US28-expressing HEK293T membranes by periplasmic extracts expressing single nanobody clones. Candidate clones are indicated with blue circles. **b**, **c** Displacement of ^125^I-CCL5 (**b**) or ^125^I-CX3CL1 (**c**) from US28 on HEK239T membranes by monovalent or bivalent nanobodies or unlabeled ligand. Monovalent nanobody: open blue circles, bivalent nanobody: filled red circles and unlabeled ligand: filled black squares. **d** Lack of displacement of ^125^I-CX3CL1 from CX3CR1-expressing HEK293T membranes by the US28 nanobodies. The data show mean ± SEM. ***P < 0.001 (unpaired *t* test)

**Fig. 5:**
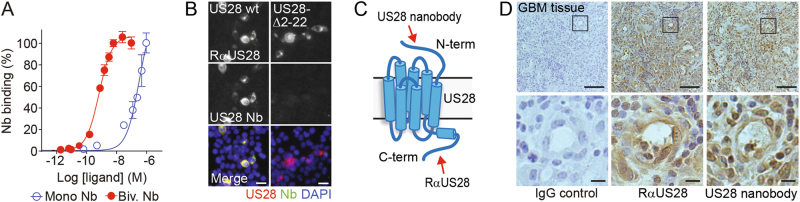
Nanobodies bind the N-terminus of US28 and detect US28 in GBM tissue. **a** Binding of monovalent and bivalent US28-nanobodies to US28-expressing HEK239T cells, as detected by flow cytometry. **b** Nanobody binding to US28 wild type or N-terminally truncated US28-Δ2–22 mutant, as determined by fluorescence microscopy. US28 is detected with rabbit-anti-US28 antibodies (RαUS28), nanobodies with anti-Myc (green) and nuclei with DAPI (blue).**c** Indication of the binding epitopes of the rabbit-anti-US28 antibodies (C-terminal) and nanobody (N-terminal) on US28. **d** Immunohistochemistry staining of US28 using bivalent US28-nanobodies. Parallel sections of GBM patient material were stained with hematoxylin (blue) and bivalent US28 nanobody or rabbit-anti-US28 antibodies (brown). IgG isotype control (IgG) was used as control. Nanobodies were detected via their Myc-tag. Scale bars represent 250 μm and 100 μm (inset)

In order to study the oncomodulatory role of the HCMV-encoded chemokine receptor US28 in more detail, we set out to generate inhibitory US28-targeting nanobodies. Phage-display libraries were generated from RNA from peripheral blood lymphocytes of immunized llama and alpaca and this library was used in multiple rounds of phage-display selection. Output clones were screened for their ability to displace the radiolabeled chemokine CCL5 (^125^I-CCL5) from US28-expressing cells (Fig. 4a). One candidate that fully inhibited the binding of radiolabeled CCL5 or CX3CL1 to US28 was designated as monovalent or Mono US28-Nb (Fig. 4b, c and Table 1). A bivalent construct of this nanobody (Biv. US28-Nb) showed a 100-fold increase in affinity as shown in competition binding (pK_i_ of 9.0 ± 0.2 for CCL5 and 9.6 ± 0.2 for CX3CL1, Fig. 4b, c and Table 1) and flow cytometry assays (p*K*_d_ of 9.2 ± 0.1, Fig. 5a and Table 1). The US28 nanobody did not inhibit binding of radiolabeled CX3CL1 to its cognate human receptor CX3CR1 (Fig. 4d), illustrating its selectivity towards US28 [33, 34]. The US28 nanobody did not bind to a US28 mutant in which the N-terminal was truncated by deletion of 21 amino acid residues (US28-∆2–22), indicating that its binding epitope involves the N-terminal residues of US28 (Fig. 5b).

**Table 1:** Pharmacological characteristics of US28 nanobodies

Assay		US28 Nb	Biv. US28 Nb
Binding affinity	p*K*_d_	6.5 ± 0.2	9.2 ± 0.1
^125^I-CCL5	Displ. (%)	104 ± 10	119^ ± ^9
	p*K*_i_	7.0 ± 0.1	9.0 ± 0.2
^125^I-CX3CL1	Displ. (%)	77 ± 1^a^	94 ± 5
	p*K*_i_	7.2 ± 0.2	9.6 ± 0.2
NF-κB reporter gene	Activity (α)	–	−0.50 ± 0.08
	pIC_50_	–	8.6 ± 0.3
InsP accumulation	Activity (α)	–	−0.53 ± 0.02
	pIC_50_	–	8.8 ± 0.1

Binding affinity in flow cytometry (p*K*_d_), radioligand displacement (%), potency (p*K*_i_ or pIC_50_) and efficacy of US28 nanobodies in HEK293T cells. Mean values ± SEM are shown

^a^Incomplete curve up to 10^−6^ M

The high affinity and selectivity of the nanobodies to the extracellular region of US28 (Fig. 5c) allows them to serve as a diagnostic tool for detecting US28 in patient material. To this end, the bivalent nanobodies were used for immunohistochemistry on parallel paraffin-embedded sections of a patient GBM tissue sample (Fig. 5d). On parallel sections, the US28 nanobody showed similar specific staining of US28 as the conventional US28 antibody that was raised against the C-terminus of the receptor [21]. Taken together, a novel US28-targeting nanobody was generated which competes for chemokine binding via binding to the N-terminus of the receptor. These nanobodies stained a similar US28-expressing cell population in GBM samples as the previously published polyclonal US28 antibody. These data demonstrate the potential of the US28 nanobodies as a high quality US28 detection tool for ex vivo and in vivo applications.

**Fig. 6:**
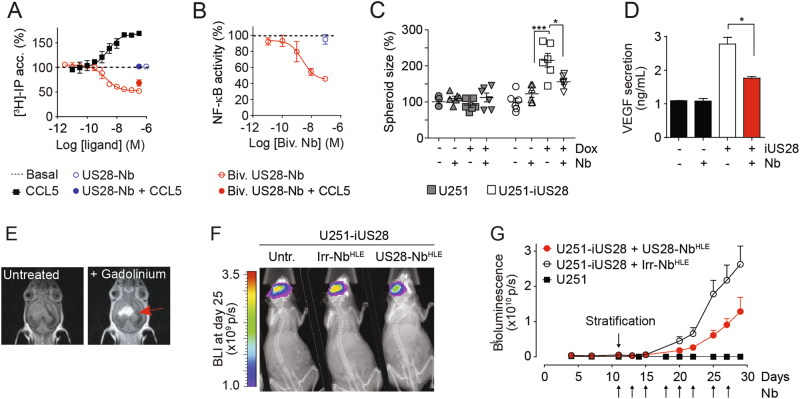
Inhibition of US28-mediated GBM-growth by US28-specific nanobodies. **a** US28-mediated accumulation of ^3^H-labeled inositol phosphates (IP) in US28-expressing HEK239T cells treated with either CCL5 alone (black squares), monovalent US28 nanobody alone (10^−6^ M, open blue circle), monovalent nanobody with CCL5 (10^−6.5^ M, filled blue circle), bivalent US28-nanobodies alone (concentration range, open red circles) or bivalent nanobody with CCL5 (10^−6,5^ M, filled red circle). CCL5 was added at 10^−7.5^ M. IP levels were plotted in percentage of basal US28-mediated IP accumulation (dashed line). **b** US28-induced NF-κB reporter gene activation upon treatment with Biv. US28-Nb (red) or Mono US28-Nb (blue) in HEK239T cells, plotted in percentage of basal US28-mediated NF-κB activation (dashed line). **c** Inhibition of US28-mediated growth of U251-iUS28 spheroids by bivalent US28-Nbs (US28-Nb, 10^−7^ M), plotted in percentages as mean ± SEM (*n* = 6 spheroids per group). **d** Secretion of VEGF from U251–iUS28 spheroids upon treatment with bivalent US28-Nbs (10^−7^ M), as detected using ELISA. **e** T1 weighed MRI scan of a brain of one of the mice carrying an orthotopic doxycycline-induced U251-iUS28 tumor before (left) and after (right) intravenous administration of the contrast agent gadolinium. The impaired blood–brain barrier function this tumor model is visualized by extravasation of gadolinium from the blood vasculature into the tumor (red arrow). **f**, **g** Growth of orthotopic GBM tumors in mice upon treatment with half-life extended (HLE) bivalent US28-nanobodies (US28-Nb^HLE^, red circles), or an irrelevant nanobody (Irr.-Nb^HLE^, open black circles). Nanobody treatment (500 μg/injection, 3 times a week, black arrows) was started upon tumor take and stratification of the mice (day 11). Pooled data of *n* = 2 with 6 mice per group per experiment. ***P < 0.001, *P < 0.05 (unpaired *t* test)

We subsequently assessed the effect of the nanobodies on US28 signaling. The CCL5-induced increase in Gα_q_ signaling associated with an accumulation of inositol phosphates (IP) was fully inhibited by the monovalent nanobody (Fig. 6a) in a concentration-dependent manner (Supplementary Fig. 4A, pIC_50_ of 7.2 ± 0.09 at a [CCL5] of 10^−7.5^ M, which equals EC_80_). Importantly, the bivalent nanobody not only antagonized the CCL5-induced signaling, but also inhibited the constitutive US28-mediated IP accumulation in a concentration-dependent manner (*α* −0.52 ± 0.02, pIC_50_ 8.8 ± 0.1, Fig. 6a). The monovalent Nb did not inhibit the basal US28 activity at a concentrations that corresponded to 10-times (10^−6^ M, Fig. 6a) or 100-times (10^−5^ M, not shown) its k_D_ value. The inverse agonistic activity of the bivalent nanobody was confirmed in an orthogonal reporter gene assay in which the bivalent nanobody, but not the monovalent nanobody, inhibited US28-induced NF-κB activity (*α* −0.50 ± 0.08 and pIC_50_ 8.6 ± 0.3, Fig. 6b). In accordance with the epitope mapping data, the nanobodies did not inhibit the signaling of the US28-∆2–22 mutant (Supplementary Fig. 4B). In addition, the bivalent nanobodies impaired US28-dependent foci growth (Supplementary Fig. 4C) and proliferation (Supplementary Fig. 4D) in NIH-3T3 cells. These data demonstrate that the monovalent US28-targeting nanobody acts as a competitive antagonist on US28, whereas the bivalent nanobody acts both as antagonist in chemokine binding and as a partial inverse agonist in US28 signaling.

### Bivalent nanobody impairs US28-enhanced GBM growth

Next, we tested the bivalent US28 nanobody in in vitro and in vivo GBM models. In U251-iUS28 cultures, the bivalent nanobody stained US28 and inhibited US28-mediated signaling and VEGF secretion (Supplementary Fig. 5A–C). Moreover, it significantly inhibited US28-mediated growth of U251-iUS28 spheroids by approximately 50% (Fig. 6c), which was accompanied by significantly reduced VEGF secretion (Fig. 6d). Treatment of these spheroids with the clinically approved VEGF scavenging antibody bevacizumab (BCM)/Avastin [35] reduced the US28-mediated growth of U251-iUS28 spheroids to a similar extent as the bivalent nanobody (Supplementary Fig. 6). However, BCM/Avastin did not affect 3D spheroid growth of non-induced iUS28-U251 cells, and there was no additional effect when BCM and the nanobody were combined.

As a next step, the functionality of the nanobody was tested in the in vivo GBM model. The blood–brain barrier is a major hurdle in drug delivery in the brain. Therefore, the integrity of the tumor vasculature was first assessed by T1 weighed MRI (Fig. 6e). The observed extravasation of the contrast agent gadolinium indicates a compromised integrity of the blood–brain barrier in these tumors, which prompted us to test our nanobodies in this model via systemic administration. Because of their small size, nanobodies display an efficient renal clearance and consequently a relatively short blood circulation time [26]. To enhance their circulation half-life, an additional albumin-binding nanobody was added to the bivalent US28-specific nanobody, creating a trivalent construct with half-life extension (designated as US28-Nb^HLE^) [36]. A bivalent nanobody directed against Pseudomonas transport protein PcrV [37] was also half-life extended and was taken along as irrelevant control (Irr-Nb^HLE^). US28-Nb^HLE^ displayed a similar potency/efficacy in inhibiting US28 signaling compared to the unmodified bivalent nanobody, while the Irr-Nb-HLE did not show binding to US28 and did not affect US28 signaling (Supplementary Fig. 7). Systemic intraperitoneal administration of mice bearing orthotopic U251-iUS28 tumors with US28-Nb^HLE^ significantly impaired tumor growth (Fig. 6f, g). This inhibition was most evident on day 25 and 27 after surgery (*p* < 0.02 and <0.04 respectively). Thus, nanobodies targeting US28 and modulating its constitutive activity have therapeutic efficacy in vivo.

**Fig. 7:**
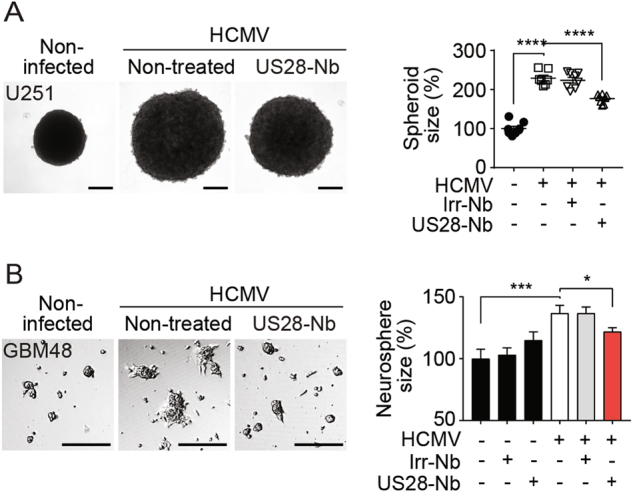
Nanobodies impair HCMV-enhanced GBM spheroid growth. **a** Spheroid growth of U251 GBM cells infected with HCMV-Merlin upon treatment with Irr-Nb or US28-Nb (*n* = 8 spheroids per group). **b** Neurosphere growth of non-infected and HCMV-infected primary GBM48 cells upon treatment with Irr-Nb or US28-Nb (*n* = > 150 neurospheres per group). All scale bars represent 250 μm. Individual spheroid- or neurosphere sizes were quantified and plotted in percentages with mean ± SEM. ****P < 0.0001, ***P < 0.001, *P < 0.05 (unpaired *t* test)

To determine the role of US28 signaling in GBM in a clinically more relevant context, U251 GBM cells (Fig. 7a) and GBM48 primary GBM cells (Fig. 7b) were infected with the clinically relevant HCMV Merlin strain and subsequently treated with either bivalent US28-Nb or Irr-Nb. While the bivalent US28-specific nanobodies showed no effect on non-infected U251 spheroids (Supplementary Figure 6) and GBM48 neurospheres (Fig. 7b), inhibition of US28 signaling by these nanobodies impaired the HCMV-mediated growth of both U251 spheroids and GBM48 neurospheres. From these data, we conclude that the constitutive signaling of the viral chemokine receptor US28 plays an important role in the oncomodulatory effect of HCMV in GBM.

## Discussion

The widespread pathogen HCMV is thought to behave as an oncomodulator in various malignancies, including GBM [3, 19, 38]. The HCMV-encoded chemokine receptor US28 has been shown to be important in virus latency, cell-to-cell dissemination, immune evasion and angioproliferative signaling and has been detected in GBM patient samples [3, 18, 39]. This makes this viral receptor a potential, foreign (non-human) target for treatment of HCMV-associated diseases, including cancer. In this study, we demonstrate an important role of HCMV/US28-mediated signaling in tumorigenesis and provide the first evidence that US28-specific nanobodies have therapeutic potential against HCMV-positive GBM.

HCMV infection is associated with enhanced angioproliferative and invasive phenotype of GBM cells [3, 19]. This was previously shown for HCMV-infected 2D cultures of U251 and U87 cells [19, 21]. In our experiments, the enhanced spheroid growth of US28-expressing U251 cells was accompanied by elevated levels of VEGF secretion. Such enhanced VEGF secretion was previously described to be a consequence of the promiscuous signaling of US28 to the transcription factors NF-kB, STAT3 and HIF-1 [19–22, 25]. Notably, elevated expression levels of VEGF are correlated with high-grade malignancy and strongly contribute to gliomagenesis [40]. The US28-accelerated tumor development of oncogenic glioma cells in vivo, supports the concept of US28 to be implicated in HCMV-mediated oncomodulation. This is in line with previous findings of US28- dependent tumor formation of non-transformed NIH-3T3 cells [22] and intestinal neoplasia in US28 transgenic mice [41].

To further substantiate the role of US28 in HCMV-associated progression of GBM, we generated nanobodies binding and inhibiting US28. Previously, inhibitory small molecules targeting US28 were developed, but these displayed micromolar potencies and insufficient specificity to permit in vivo application [42]. The enhanced binding affinity and potency in ligand displacement of the bivalent nanobody is likely a consequence of avidity interactions between the nanobodies and US28. As previously observed, similar multivalent nanobody/antibody formats generated enhanced potencies because of such avidity interactions [30, 31, 43]. More interestingly, while the monovalent nanobody antagonized CCL5-dependent modulation of US28 activity, the bivalent US28 nanobody also displayed inverse agonistic properties, reducing constitutive US28 signaling. Such inverse agonistic activity of bivalent nanobodies has also been observed previously for the chemokine receptors CXCR4 [30], CXCR7 [31] and CXCR2 [29]. The molecular mechanism associated with this inverse agonistic activity is currently unclear and remains to be studied in further detail. Chemokines (e.g., CCL2 and CCL5) can exacerbate the US28-mediated phenotype as indicated by previous studies in US28 transgenic mice and in vitro GBM cell studies [19, 41]. The functional differences between the monovalent and bivalent nanobodies could be useful for dissecting the role of constitutive and chemokine-dependent US28 signaling.

In glioblastoma, enhanced VEGF levels contribute to gliomagenesis [40]. While the US28 nanobodies impaired the US28-enhanced VEGF secretion and US28-accelerated tumor phenotype, no additional effect was observed by BCM/Avastin. This suggests that in these cells, the oncomodulatory phenotype of US28 is mainly VEGF mediated. However, other factors could also still contribute to this phenotype.

HCMV is often maintained in a latent state in a subset of cells (e.g., cells of myeloid lineage), in which very few HCMV-encoded proteins, including US28, are expressed and affect cellular and immunological processes [44, 45]. Although US28 is expressed during lytic HCMV replication, it also promotes a latent state in hematopoietic progenitor cells by suppressing immediate early (IE) gene transcription and subsequent virus production [18]. Because the current benchmark anti-HCMV agent valganciclovir does not target latent virus and is associated with antiviral-resistance and off-target side effects, alternative HCMV targets like US28 and therapeutics like US28-directed nanobodies could form a potential approach to combat HCMV-related diseases, like GBM. Despite the high potencies of these bivalent nanobodies, the efficacy is limited to 50% of the constitutive activity of US28. This allows opportunities for improving the efficacy of such nanobodies via, for example, engineering of the nanobodies through addition of functional moieties, including toxins [46] and photosensitizers [47]. Recently, a modified CX3CL1 with enhanced specificity towards US28 compared to its natural receptor CX3CR1, was fused to a biological exotoxin from Pseudomonas [48]. This chemokine-toxin fusion protein efficiently inhibited viral replication in vivo by specific killing of US28-expressing and latently infected cells. Similar but nanobody-based toxin fusion proteins would be more selective. Alternatively, the nanobodies could serve as specific targeting agents for the delivery of cytotoxic agents into US28-expressing cells as a potential treatment of CMV positive solid tumors.

The high affinity of these nanobodies towards US28 also allows them to be used as detection tools. US28 protein expression in GBM was detected earlier by immunohistochemistry using a polyclonal antibody directed against the C-terminal tail of the receptor [19, 21]. In this study US28 expression was confirmed using a monoclonal nanobody format that targeted a distinct, extracellular, epitope, which further substantiates the presence of HCMV and US28 in GBM. US28 nanobodies have the potential to serve as a high quality US28 detection tool for ex vivo and in vivo applications. Being one of the few markers of a latent HCMV-infection [44], the US28 nanobodies could be used to detect latently infected cells or so-called latent HCMV-reservoirs. Since HCMV is also detected in other malignancies such as colorectal, lung, breast and prostate tumors [3], the US28 nanobodies could be used to evaluate the presence and role of US28 in other HCMV-related pathologies as well. For example, US28 expression was recently detected in renal allografts and was suggested to play a role in renal dysfunction after organ transplantation [5]. Because the nanobodies recognize the extracellular and therefore accessible parts of US28, they could potentially be used for imaging in patients by for example positron emission tomography. Hence, the US28-targeting nanobodies can potentially be applied for both patient-stratification (i.e., diagnostic) and therapeutic intervention.

Taken together, this study further emphasizes the oncomodulatory role of US28 and shows the consequence of the expression of the HCMV-encoded chemokine receptor US28 in GBM in vivo. Furthermore, we have generated llama-derived nanobodies that are highly selective and act as inverse agonists inhibiting the constitutive oncomodulatory activity of US28. These molecules are promising tools for studying US28 biology, non-invasive imaging of latent and active HCMV infections and have potential in GBM therapy in HCMV-positive patients.

## Materials and methods

### Reagents

Human chemokines CCL5 and CX3CL1 (PeproTech, Rocky Hill, NJ, USA). Dulbecco’s modified Eagle’s medium (DMEM), Dulbecco’s Phosphate-buffered saline (D-PBS), trypsin-EDTA and Poly-L-lysine solution (Sigma-Aldrich, Saint Louis, MO, USA. Fetal bovine serum (FBS) and penicillin/streptomycin (PAA Laboratories GmbH, Cölbe, Germany). ^125^I-Na and myo-[2-^3^H] inositol (1 mCi/ml) (Perkin Elmer Life Sciences, Waltham, MA, USA). Mouse anti-cytomegalovirus (immediate early antigen; MAB180R, Merck Millipore, Billerica, MA, USA). Polyclonal rabbit-anti-US28 antibodies were generated by Covance (Princeton, NJ, USA) as described previously [41]. Anti-Myc tag (clone 9B11, #2276, Cell Signaling Technology, Danvers, MA, USA). Horseradish peroxidase (HRP)-conjugated antibodies (1706515 and 1706516, Bio-Rad Laboratories, Hercules, CA, USA). Alexa Fluor-conjugated antibodies (A11001, A11003, A11008 and A11010, Thermo Fisher Scientific, Waltham, MA, USA).

### Cell lines and cell culture

HEK293T, NIH 3T3, U251-MG and,COS7 cells were previously described [21, 42]. The U251 cell line was authenticated by STR profiling (Baseclear B.V., Leiden, The Netherlands). NIH 3T3 cells stably expressing US28-WT were described previously [22]. Cell lines with constitutive Fluc/mCherry (FM) expression and/or inducible US28 expression (U251-iUS28) were generated by lentiviral transduction. US28 expression was induced by 1 μg/mL doxycycline (D9891, Sigma-Aldrich). HFFF TR cells [49] were kindly provided by Dr. Richard J. Stanton. All cell lines were mycoplasma negative (PCR testing, Microbiome, Amsterdam, The Netherlands). The primary GBM48 cells were obtained from a patient with glioblastoma multiform IV (GBM) at the Karolinska Institute and were cultured in DMEM/F12 medium with 10% (v/v) heat-inactivated FBS.

### Generation of spheroids and neurospheres

Spheroids were generated by seeding 3·10^4^ U251 cells per well in a 96-well hanging drop plate (3D Biomatrix, Ann Arbor, MI, USA). After 48 h of sedimentation, spheroids were transferred to 6-well plates coated with 0.75% agarose in normal growth medium. Spheroids were imaged after 72 h. GBM48 neurospheres were generated as described previously [50]. GBM48 cells were infected with HCMV 24 h before neurosphere formation and neurospheres were imaged 7 days post infection.

### Microscopy imaging

Immunofluorescence staining and microscopy imaging were performed as previously [21, 51]. All images were obtained using an FSX-100 microscope (Olympus, Tokyo, Japan) at either 4 × (spheroids or neurospheres) or ×20 magnification (cells).

### Quantitative PCR

RNA was isolated 7 days post-infection using TRIzol (Life Technologies, Inc.). All qPCR materials were from Bio-Rad Laboratories (Hercules, CA, USA). qPCR was performed using 20 ng cDNA (transcribed using iScript Supermix), iQ SYBR Green Supermix, the MyiQ system and the following primers (150 nM each): *US28* 5′-TCCATGGGCAACTTCTTGGT-3′ and 5′-TCGCCGGAGCATTGAATC-3′ and *β-actin* 5′-AGAGCTACGAGCTGCCTGAC-3′ and 5′-GGATGCCACAGGACTCCA-3′. Data analysis was performed using Bio-Rad iQ5 software.

### Molecular cloning, transfection and transduction

The pcDEF3 plasmid encoding US28-WT (from HCMV strain VHL/E, GenBank: L20501.1) was described previously [52]. HEK293T and COS7 cells were transfected using linear 25 kDa polyethylenimine (PEI; Polysciences Inc., Warrington, PA, USA) [52]. For lentiviral transduction, the US28 DNA was subcloned in the pLenti6.3/To/V5-DEST vector. Lentivirus was produced for 48 h upon co-transfection of HEK293T cells with CMV Fluc-IRES-mCherry, US28-VHL/E pLenti6.3/To/V5-DEST or pLenti3.3/TR (Thermo Scientific) together with pRSV-REV, pMDLg/pRRE and pMD2.g packaging vectors. Lentivirus solution was cleared by centrifugation and filtration and cells were transduced overnight.

### HCMV infection

The HCMV strain TB40/E WT and the deletion mutant TB40/E ∆US28 were previously described [53]. HCMV strain Merlin was generated from a bacterial artificial chromosome BAC pAL2157. This is BAC pAL1498 [49] in which eGFP was linked to the IE2 gene using a P2A linker. SW102 *E. coli* containing BAC pAL2157 were kindly provided by Dr. Richard J. Stanton. BAC DNA was isolated using NucleoBond Xtra BAC kit (Macherey Nagel, Düren, Germany). Virus production was initiated by electroporation of BAC DNA into HFFF TR cells using the Amaxa Nucleofector and the basic fibroblast nucleofector kit (Lonza, Basel, Switserland). Subsequent virus productions in HFFF TR cells were started with an infection at MOI 0.02 and titres were determined after 3 days using immediate early antigen staining. U251 and GBM48 GBM cells were infected at MOI 3.

### Generating nanobodies

US28 cDNA encoded by VHL/E strain (GenBank: L20501.1) was subcloned from pcDEF_3_ to pVAX1 (Thermo Fisher Scientific). One llama and one alpaca were immunized 4 times with pVAX1-US28 DNA, followed by boost immunizations with HEK293T cells expressing US28. Subsequently, nanobody phage-libraries were generated as previously [30] and phage-display selections were performed on US28-expressing virus-like particles (Integral Molecular, Philadelphia, PA, USA) and US28-expressing COS7 membrane extracts. Nanobody clones were screened for displacement of ^125^I-CCL5 (100 pM) using *E. coli* periplasmic extracts. Hits showed a 3xSD-value reduction in ligand binding. A bivalent nanobody construct was generated via a genetic head-to-tail fusion of two identical nanobodies, separated by a 35 GS-linker (i.e., 7 GGGGS-repeats). Nanobodies were produced and purified from BL21 *E. coli* [51]. Half-life extended nanobodies were produced in *Pichia Pastoris* strain XL-33 [31].

### Binding assays

Displacement and cell-surface binding of ^125^I-labeled chemokines CCL5 or CX3CL1 [42, 52] was analyzed using a Competitive One-Site binding fit and Competitive One-Site FitLogIC50 binding fit respectively. Displacement of radiolabeled chemokines with 10^−6^ M of unlabeled CX3CL1 was taken as full displacement. Flow cytometry was performed as described previously [51].

### US28 signaling assays

Activation of phospholipase C was determined by quantification of the [^3^H]-inositol phosphates (InsP) [21]. NFκB-luciferase reporter gene assays were performed in HEK293T cells [52] and luminescence (1 s per well) was measured with a Mithras LB940 multilabel plate reader (Berthold Technologies). VEGF levels were measured in conditioned medium by ELISA using the Quantikine human-VEGF ELISA kit (R&D systems) [25]. In all VEGF ELISA assays, the lower detection limit was 15 pg/ml.

### Foci formation and proliferation assay

The US28-dependent formation and growth of foci was assessed in NIH 3T3 cells [21, 54]. Methylene blue-stained foci was quantified using ImageJ software. Cell proliferation of NIH 3T3 cells was quantified by total protein yield as determined by BCA assay (Bio-Rad).

### Immunohistochemistry

Paraffin-embedded animal (8 μm) and patient (5 μm) tissue sections were deparaffinized in tissue clear (Sakura) and rehydrated via a graded ethanol series. Antigen retrieval was performed using a high pressure cooker (Decloaking Chamber NxGEN) in DIVA decloacker (Biocare Medical) solution (12 min at 95 °C). Hereafter, incubation with pepsin (Sigma-Aldrich) (5 min at 37 °C), peroxidazed-1 (Biocare Medical) (3 min at 20 °C), background sniper (Biocare Medical) (15 min at 20 °C), and protein block (DAKO) (10 min at 20 °C) was performed. Primary antibody incubation with polyclonal rabbit anti-US28, 1:500 for animal tissue and 1:700 for GBM patient tissue (Covance) at 4 °C was performed overnight. MACH2 Universal HRP-Polymer Detection (Biocare Medical) served as secondary antibody. Nanobody staining was performed using similar methods: bivalent US28-Nb incubation overnight was followed by incubation with 1:500 mouse anti-Myc antibody (Cell Signaling) (1 h at 20 °C). From here on, Tyramide Signal Amplification (TSA) was used following manufacturers protocol. Sections were developed using 3,3′-diaminobenzidine (DAB) (Biocare Medical) and counterstained with aqua hematoxylin (Innovex). Finally, sections were dehydrated via graded ethanol series and xylene, and mounted using DPX neutral mounting medium (KliniPath). The study was approved by the Stockholm’s Regional ethical committee (permission number: 2008/628-31/2) in Sweden.

### Animal model studies

All animal experiments were conducted in compliance with Dutch Law on animal experimentation and the European Community Council Directive 2010/63/EU for laboratory animal care and approved by the animal experimentation commission of the VU University medical center. The required sample sizes were calculated based on in vitro data and a small in vivo pilot using a two-sided t- test, significance level (Type I error) of 0.05, power of 0.9 and assuming a 50% difference between sample means and a 20% standard deviation in both groups. 6 weeks old female athymic nude mice (Harlan/Envigo, Horst, The Netherlands) were kept as described previously [55]. With the exception of the initial US28 comparison study (3 mice per group), studies were performed with 6 mice per group. Stereotactic injections were performed with 5·10^5^ cells as described previously [55]. Mice were monitored daily and tumor development was monitored twice weekly using an IVIS/CCD camera (Caliper Life Sciences, Waltham, MA, USA) or In-Vivo Extreme imager (Bruker, Billerica, MA, USA) upon intraperitoneal injection of _D_-luciferin (150 mg/kg, GoldBio, Olivette, MO, USA). Mice were fed sucrose water (5% w/v) or doxycycline/sucrose-water (2 mg/mL, 5% w/v). Mice were stratified by tumor size and treated three times per week with PBS or nanobodies (500 μg) via intraperitoneal injections. No blinding was used. Mice were killed upon >15% weight loss, blood was drawn via cardiac puncture and brains were formalin-fixed.

### Magnetic resonance imaging

Magnetic resonance imaging (MRI) was performed using a preclinical PET-MRI system (Nanoscan system, Mediso, Budapest, Hungary) [55]. A T1 weighed scan was performed before and after injection of 750 μmol of the MRI contrast agent Gadolinium (Dotarem) in the tail vein using a cannula. MRI images were analyzed using MIPAV software (Medical Image Processing, Analysis, and Visualization, version 7.2.0).

### Statistical analyses

Unless indicated otherwise, all data represented three independent experiments, each performed in triplicates. Data and error bars represent mean ± SEM. Acquisition and analysis of data regarding spheroids, neurospheres and bioluminescence was blinded. Graphs and statistical analyses were performed with Prism 6 (GraphPad software Inc. San Diego, CA, USA). Groups were compared using Student’s *t* test (two-tailed, significance level of *α* = 0.05) or ANOVA analyses with multiple comparisons test and Tukey correction. Normality of data was determined using the D’Agostino–Pearson omnibus test. Equality of variance was confirmed with an *F* test. No data were excluded.

## Electronic supplementary material

Supplementary Information

## References

[1] Ostrom QT, Gittleman H, Fulop J, Liu M, Blanda R, Kromer C (2015). CBTRUS Statistical Report: Primary Brain and Central Nervous System Tumors Diagnosed in the United States in 2008-2012. Neuro Oncol.

[2] Bush NA, Chang SM, Berger MS (2016). Current and future strategies for treatment of glioma. Neurosurg Rev.

[3] Vischer HF, Siderius M, Leurs R, Smit MJ (2014). Herpesvirus-encoded GPCRs: neglected players in inflammatory and proliferative diseases?. Nat Rev Drug Discov.

[4] Sinclair JH, Reeves MB (2013). Human cytomegalovirus manipulation of latently infected cells. Viruses.

[5] Lollinga WT, de Wit RH, Rahbar A, Vasse GF, Davoudi B, Diepstra A (2016). Human cytomegalovirus-encoded receptor US28 is expressed in renal allografts and facilitates viral spreading in vitro. Transplantation.

[6] Cobbs CS, Harkins L, Samanta M, Gillespie GY, Bharara S, King PH (2002). Human cytomegalovirus infection and expression in human malignant glioma. Cancer Res.

[7] Bhattacharjee B, Renzette N, Kowalik TF (2012). Genetic analysis of cytomegalovirus in malignant gliomas. J Virol.

[8] Cobbs C (2014). Response to “Human cytomegalovirus infection in tumor cells of the nervous system is not detectable with standardized pathologico-virological diagnostics”. Neuro Oncol.

[9] Taha MS, Abdalhamid BA, El-Badawy SA, Sorour YM, Almsned FM, Al-Abbadi MA (2016). Expression of cytomegalovirus in glioblastoma multiforme: Myth or reality?. Br J Neurosurg.

[10] Baryawno N, Rahbar A, Wolmer-Solberg N, Taher C, Odeberg J, Darabi A (2011). Detection of human cytomegalovirus in medulloblastomas reveals a potential therapeutic target. J Clin Investig.

[11] Stragliotto G, Rahbar A, Solberg NW, Lilja A, Taher C, Orrego A (2013). Effects of valganciclovir as an add-on therapy in patients with cytomegalovirus-positive glioblastoma: a randomized, double-blind, hypothesis-generating study. Int J Cancer.

[12] Hadaczek P, Ozawa T, Soroceanu L, Yoshida Y, Matlaf L, Singer E (2013). Cidofovir: a novel antitumor agent for glioblastoma. Clin Cancer Res.

[13] Mitchell DA, Batich KA, Gunn MD, Huang MN, Sanchez-Perez L, Nair SK (2015). Tetanus toxoid and CCL3 improve dendritic cell vaccines in mice and glioblastoma patients. Nature.

[14] Rahbar A, Stragliotto G, Orrego A, Peredo I, Taher C, Willems J (2012). Low levels of Human Cytomegalovirus Infection in Glioblastoma multiforme associates with patient survival; -a case-control study. Herpesviridae.

[15] Soderberg-Naucler C, Rahbar A, Stragliotto G (2013). Survival in patients with glioblastoma receiving valganciclovir. N Engl J Med.

[16] Peng C, Wang J, Tanksley JP, Mobley BC, Ayers GD, Moots PL (2016). Valganciclovir and bevacizumab for recurrent glioblastoma: A single-institution experience. Mol Clin Oncol.

[17] Dziurzynski K, Chang SM, Heimberger AB, Kalejta RF, McGregor Dallas SR, Smit M (2012). Consensus on the role of human cytomegalovirus in glioblastoma. Neuro Oncol.

[18] Humby MS, O’Connor CM (2016). Human cytomegalovirus us28 is important for latent infection of hematopoietic progenitor cells. J Virol.

[19] Soroceanu L, Matlaf L, Bezrookove V, Harkins L, Martinez R, Greene M (2011). Human cytomegalovirus US28 found in glioblastoma promotes an invasive and angiogenic phenotype. Cancer Res.

[20] Maussang D, Langemeijer E, Fitzsimons CP (2009). Stigter-van Walsum M, Dijkman R, Borg MK, et al. The human cytomegalovirus-encoded chemokine receptor US28 promotes angiogenesis and tumor formation via cyclooxygenase-2. Cancer Res.

[21] Slinger E, Maussang D, Schreiber A, Siderius M, Rahbar A, Fraile-Ramos A (2010). HCMV-encoded chemokine receptor US28 mediates proliferative signaling through the IL-6-STAT3 axis. Sci Signal.

[22] Maussang D, Verzijl D, van Walsum M, Leurs R, Holl J, Pleskoff O (2006). Human cytomegalovirus-encoded chemokine receptor US28 promotes tumorigenesis. Proc Natl Acad Sci USA.

[23] O’Hayre M, Vazquez-Prado J, Kufareva I, Stawiski EW, Handel TM, Seshagiri S (2013). The emerging mutational landscape of G proteins and G-protein-coupled receptors in cancer. Nat Rev Cancer.

[24] Streblow DN, Soderberg-Naucler C, Vieira J, Smith P, Wakabayashi E, Ruchti F (1999). The human cytomegalovirus chemokine receptor US28 mediates vascular smooth muscle cell migration. Cell.

[25] de Wit RH, Mujic-Delic A, van Senten JR, Fraile-Ramos A, Siderius M, Smit MJ (2016). Human cytomegalovirus encoded chemokine receptor US28 activates the HIF-1alpha/PKM2 axis in glioblastoma cells. Oncotarget.

[26] Mujic-Delic A, de Wit RH, Verkaar F, Smit MJ (2014). GPCR-targeting nanobodies: attractive research tools, diagnostics, and therapeutics. Trends Pharmacol Sci.

[27] Burg JS, Ingram JR, Venkatakrishnan AJ, Jude KM, Dukkipati A, Feinberg EN (2015). Structural biology. Structural basis for chemokine recognition and activation of a viral G protein-coupled receptor. Science.

[28] Manglik A, Kobilka BK, Steyaert J (2017). Nanobodies to study G protein-coupled receptor structure and function. Annu Rev Pharmacol Toxicol.

[29] Bradley ME, Dombrecht B, Manini J, Willis J, Vlerick D, De Taeye S (2015). Potent and efficacious inhibition of CXCR2 signaling by biparatopic nanobodies combining two distinct modes of action. Mol Pharmacol.

[30] Jahnichen S, Blanchetot C, Maussang D, Gonzalez-Pajuelo M, Chow KY, Bosch L (2010). CXCR4 nanobodies (VHH-based single variable domains) potently inhibit chemotaxis and HIV-1 replication and mobilize stem cells. Proc Natl Acad Sci USA.

[31] Maussang D, Mujic-Delic A, Descamps FJ, Stortelers C, Vanlandschoot P, Stigter-van Walsum M (2013). Llama-derived single variable domains (nanobodies) directed against chemokine receptor CXCR7 reduce head and neck cancer cell growth in vivo. J Biol Chem.

[32] Wurdinger T, Badr C, Pike L, de Kleine R, Weissleder R, Breakefield XO (2008). A secreted luciferase for ex vivo monitoring of in vivo processes. Nat Methods.

[33] Gao JL, Murphy PM (1994). Human cytomegalovirus open reading frame US28 encodes a functional beta chemokine receptor. J Biol Chem.

[34] Kledal TN, Rosenkilde MM, Schwartz TW (1998). Selective recognition of the membrane-bound CX3C chemokine, fractalkine, by the human cytomegalovirus-encoded broad-spectrum receptor US28. FEBS Lett.

[35] Kim KJ, Li B, Winer J, Armanini M, Gillett N, Phillips HS (1993). Inhibition of vascular endothelial growth factor-induced angiogenesis suppresses tumour growth in vivo. Nature.

[36] Tijink BM, Laeremans T, Budde M, Stigter-van Walsum M, Dreier T, de Haard HJ (2008). Improved tumor targeting of anti-epidermal growth factor receptor Nanobodies through albumin binding: taking advantage of modular Nanobody technology. Mol Cancer Ther.

[37] De Tavernier E, Detalle L, Morizzo E, Roobrouck A, De Taeye S, Rieger M (2016). High throughput combinatorial formatting of pcrv nanobodies for efficient potency improvement. J Biol Chem.

[38] Harkins L, Volk AL, Samanta M, Mikolaenko I, Britt WJ, Bland KI (2002). Specific localisation of human cytomegalovirus nucleic acids and proteins in human colorectal cancer. Lancet.

[39] Noriega VM, Gardner TJ, Redmann V, Bongers G, Lira SA, Tortorella D (2014). Human cytomegalovirus US28 facilitates cell-to-cell viral dissemination. Viruses.

[40] Lamszus K, Ulbricht U, Matschke J, Brockmann MA, Fillbrandt R, Westphal M (2003). Levels of soluble vascular endothelial growth factor (VEGF) receptor 1 in astrocytic tumors and its relation to malignancy, vascularity, and VEGF-A. Clin Cancer Res.

[41] Bongers G, Maussang D, Muniz LR, Noriega VM, Fraile-Ramos A, Barker N (2010). The cytomegalovirus-encoded chemokine receptor US28 promotes intestinal neoplasia in transgenic mice. J Clin Investig.

[42] Casarosa P, Menge WM, Minisini R, Otto C, van Heteren J, Jongejan A (2003). Identification of the first nonpeptidergic inverse agonist for a constitutively active viral-encoded G protein-coupled receptor. J Biol Chem.

[43] Steensgaard J, Liu BM, Cline GB, Moller NP (1977). The properties of immune complex-forming systems. A new theoretical approach. Immunology.

[44] Mason GM, Jackson S, Okecha G, Poole E, Sissons JG, Sinclair J (2013). Human cytomegalovirus latency-associated proteins elicit immune-suppressive IL-10 producing CD4(+) T cells. PLoS Pathog.

[45] Soderberg-Naucler C, Fish KN, Nelson JA (1997). Reactivation of latent human cytomegalovirus by allogeneic stimulation of blood cells from healthy donors. Cell.

[46] Li YM, Hall WA (2010). Targeted toxins in brain tumor therapy. Toxins.

[47] van Driel PB, Boonstra MC, Slooter MD, Heukers R, Stammes MA, Snoeks TJ (2016). EGFR targeted nanobody-photosensitizer conjugates for photodynamic therapy in a pre-clinical model of head and neck cancer. J Control Release.

[48] Krishna BA, Spiess K, Poole EL, Lau B, Voigt S, Kledal TN (2017). Targeting the latent cytomegalovirus reservoir with an antiviral fusion toxin protein. Nat Commun.

[49] Stanton RJ, Baluchova K, Dargan DJ, Cunningham C, Sheehy O, Seirafian S (2010). Reconstruction of the complete human cytomegalovirus genome in a BAC reveals RL13 to be a potent inhibitor of replication. J Clin Investig.

[50] Caretti V, Sewing AC, Lagerweij T, Schellen P, Bugiani M, Jansen MH (2014). Human pontine glioma cells can induce murine tumors. Acta Neuropathol.

[51] de Wit RH, Heukers R, Brink HJ, Arsova A, Maussang D, Cutolo P (2017). CXCR4-specific nanobodies as potential therapeutics for WHIM syndrome. J Pharmacol Exp Ther.

[52] Casarosa P, Bakker RA, Verzijl D, Navis M, Timmerman H, Leurs R (2001). Constitutive signaling of the human cytomegalovirus-encoded chemokine receptor US28. J Biol Chem.

[53] Langemeijer EV, Slinger E, de Munnik S, Schreiber A, Maussang D, Vischer H (2012). Constitutive beta-catenin signaling by the viral chemokine receptor US28. PloS ONE.

[54] Burger M, Burger JA, Hoch RC, Oades Z, Takamori H, Schraufstatter IU (1999). Point mutation causing constitutive signaling of CXCR2 leads to transforming activity similar to Kaposi’s sarcoma herpesvirus-G protein-coupled receptor. J Immunol.

[55] Jansen MH, Lagerweij T, Sewing AC, Vugts DJ, van Vuurden DG, Molthoff CF (2016). Bevacizumab targeting diffuse intrinsic pontine glioma: results of 89Zr-bevacizumab PET imaging in brain tumor models. Mol Cancer Ther.

