# Supplementary material for: The constitutive activity of the virally encoded chemokine receptor US28 accelerates glioblastoma growth
Source: Oncogene. 2018 Apr 30;37(30):4110–21. doi: 10.1038/s41388-018-0255-7 (PMC6062493; doi:10.1038/s41388-018-0255-7)
Supplement: Supplementary file 1 — Supplementary Information [file 41388_2018_255_MOESM1_ESM.docx]

**Supplementary Information**

The constitutive activity of the virally-encoded chemokine receptor US28 accelerates glioblastoma growth

Raimond Heukers, Raymond H. de Wit, Tian Shu Fan, Jeffrey R. van Senten, Timo W.M. De Groof, Maarten P. Bebelman, Tonny Lagerweij, Joao Vieira, Sabrina M. de Munnik, Laura Smits-de Vries, Jody van Offenbeek, Afsar Rahbar, Diane van Hoorick, Cecilia Söderberg- Naucler, Thomas Würdinger, Rob Leurs, Marco Siderius, Henry F. Vischer, Martine J. Smit

**
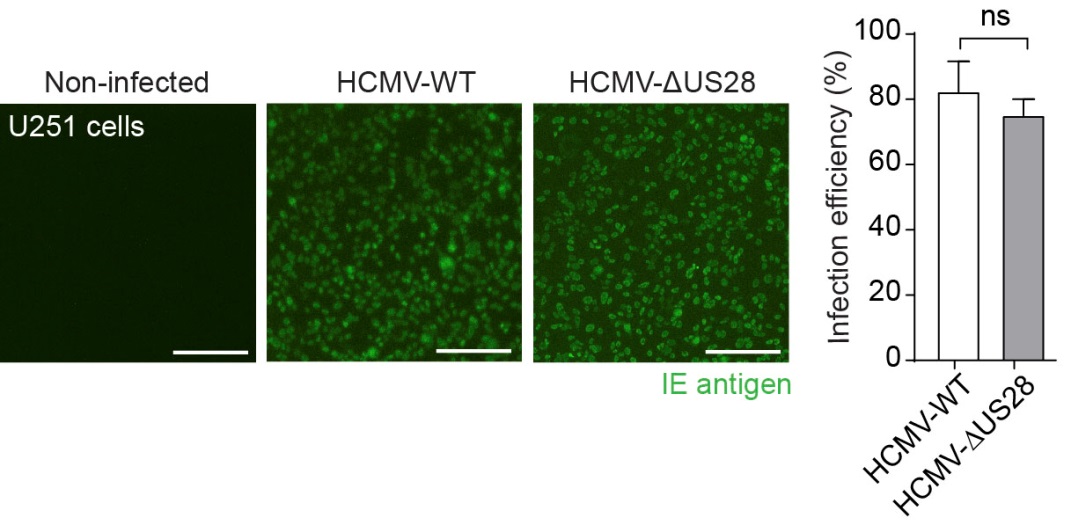
**

**Supplementary Figure 1** Infection rate of HCMV strain TB40/E wild type and HCMV-∆US28 in U251 cells. Cells quantified for immediate early antigen (IE) expression. Scale bars represent 250 μm.


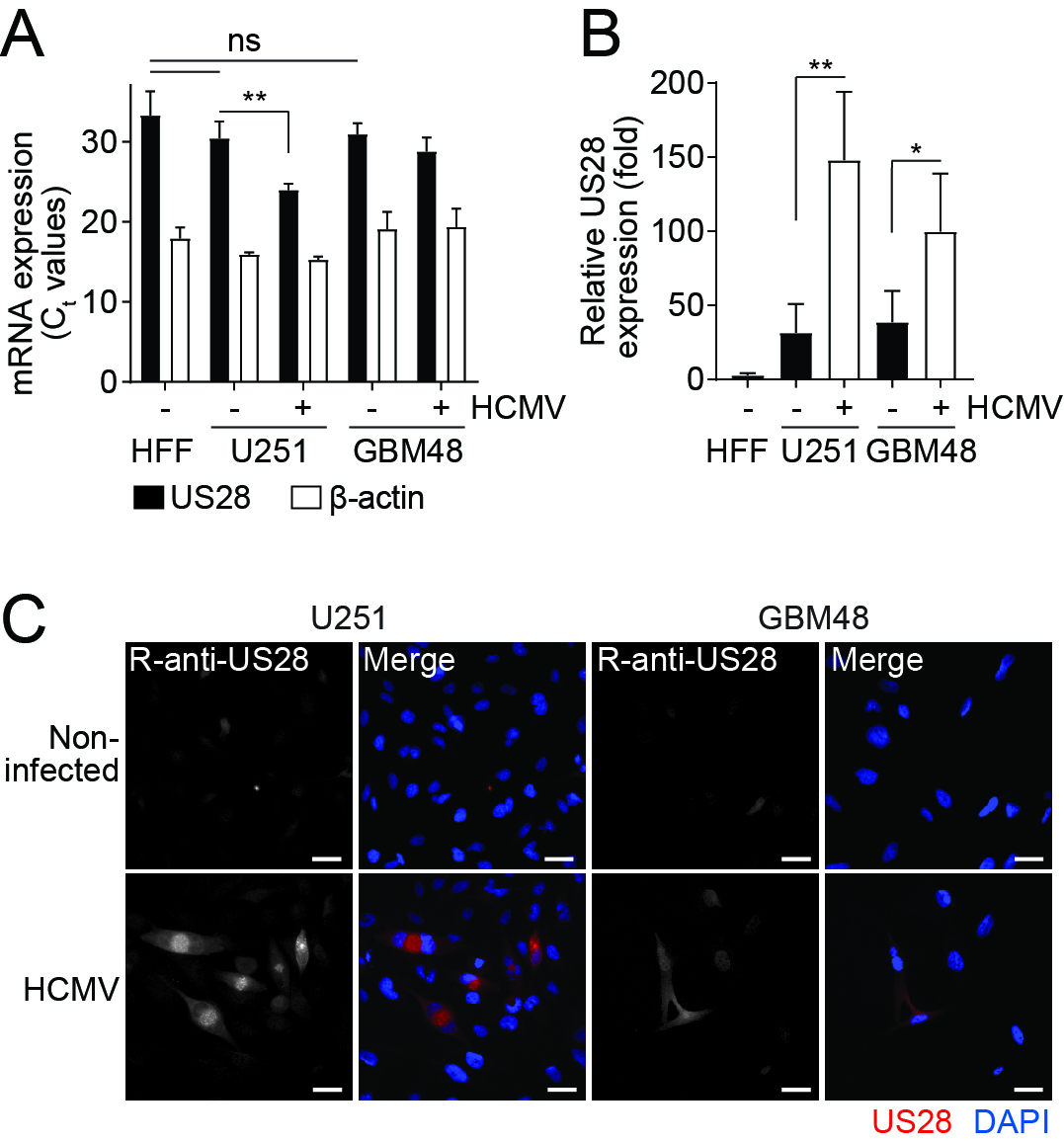


**Supplementary Figure 2** Expression of US28 upon HCMV infection of glioblastoma cells. (A) qPCR-derived Ct-values of US28 and β-actin control mRNA in non-infected or HCMV(Merlin)-infected U251 or GBM48 cells and non-infected HFF cells as control. (B) Relative US28 mRNA expression, plotted in fold increase (representing 2^-∆∆Ct^) compared to HCMV-negative HFF cells. * P < 0.05 and ** P < 0.02 (unpaired t-test). (C) US28 protein expression in non-infected or HCMV(Merlin)-infected U251 (left) or GBM48 (right) cells, as visualized by immunofluorescence microscopy imaging. Scale bars represent 20 μm.

**
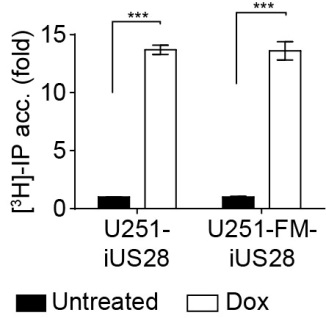
**

**Supplementary Figure 3.** U251-FM-iUS28 cells show similar properties as their original counterparts. PLC activation in U251-iUS28 or U251-FM-iUS28 cells upon induction of US28 expression. PLC activation was determined by the fold increase in inositol phosphate ([^3^H]-IP) accumulation as compared non-induced cells. *** P < 0.001 (unpaired t-test).


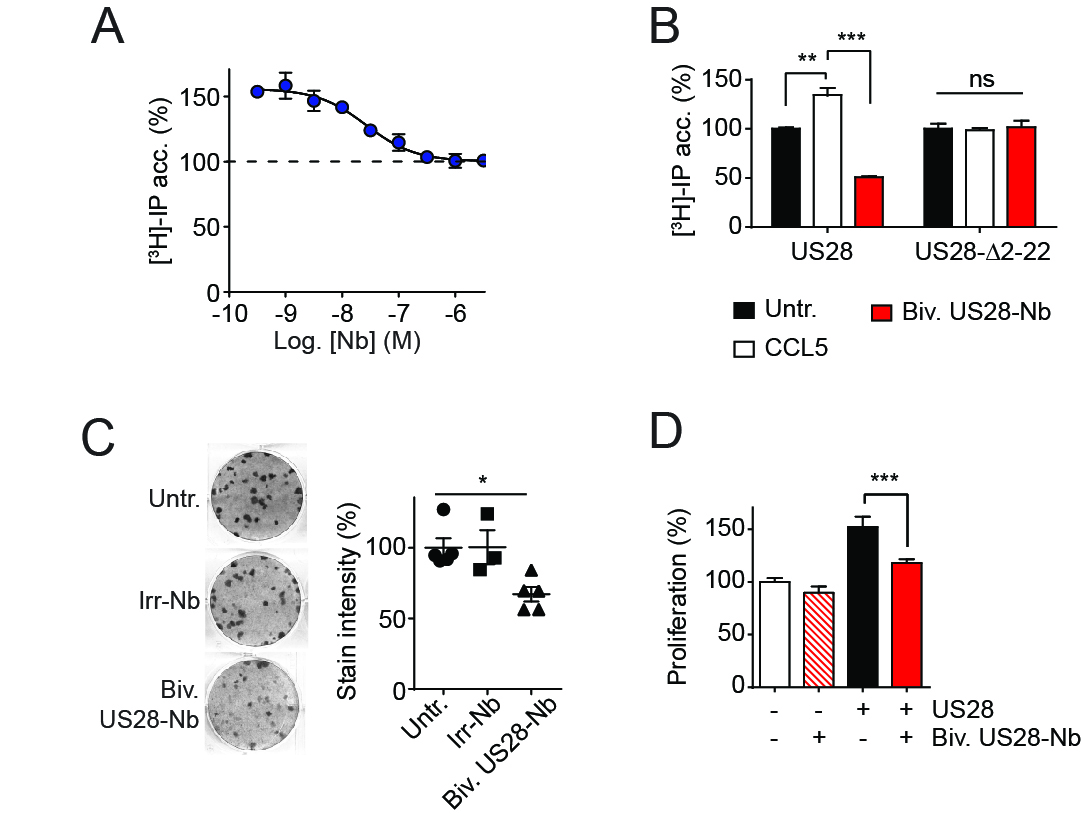


**Supplementary Figure 4** Nanobodies impair ligand-induced and constitutive US28 signaling. (A) Inhibition of CCL5-induced signaling towards PLC by Mono Nb. CCL5-induced accumulation of inositol phosphates ([^3^H]-IP) was determined in HEK293T cells treated with 10^-7.5^ M (EC_80_) of CCL5 and a concentration range of Mono Nb. Basal US28-induced IP accumulation was plotted as 100% (dashed line). (B) Effect of CCL5 (10^-8^ M) or bivalent US28-nanobodies on US28-mediated IP accumulation in HEK239T cells expressing US28 wild type or the US28-∆2-22 mutant. (C) Inhibition of US28-mediated NIH-3T3 foci growth by bivalent US28-nanobodies. Foci were allowed to form during an incubation of 14 days in which medium with nanobodies was replaced every 3 days. Growth of individual foci was measured by determining methylene blue stain intensity. Plotted are averages of individual experiments with mean ± SEM. (D) Inhibition of US28-induced proliferation of NIH-3T3 cells by Biv. US28-Nb. Cells were grown for 7 days in which medium with nanobodies was refreshed every 3 days. Proliferation was quantified by total protein determination in cell lysates. Unless indicated otherwise, all nanobody treatments were done with a concentration of 10^-7^ M. *** P < 0.001, ** P < 0.01, * P < 0.05 (unpaired t-test).

**
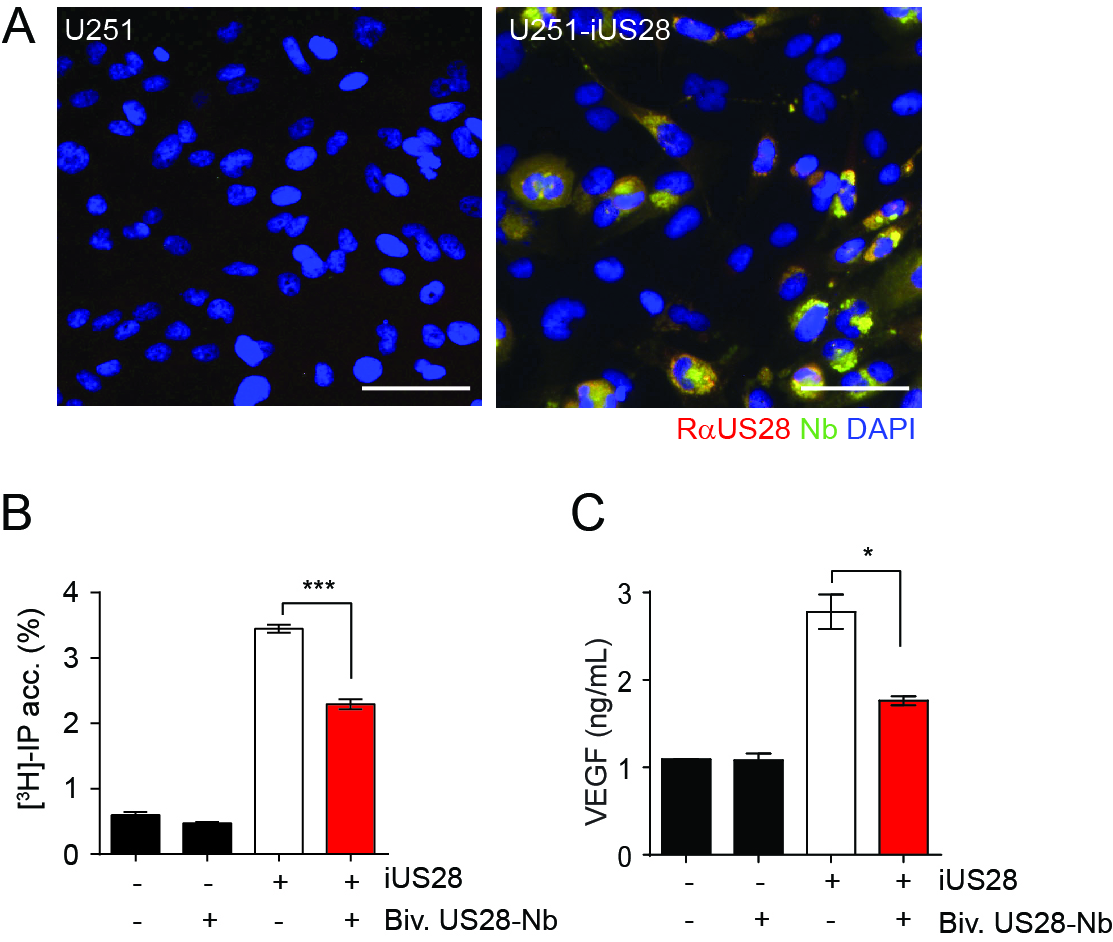
**

**Supplementary Figure 5** US28-specific nanobodies inhibit US28 in 2D U251 GBM cultures. (A) Binding of US28-Nb to US28 in U251-iUS28 cells as determined by fluorescence microscopy imaging. (B) Inhibition of US28-mediated inositol phosphate (IP) accumulation in U251-iUS28 cells upon treatment with bivalent US28-Nb. (C) Inhibition of US28-induced secretion of VEGF from U251-iUS28 cells.

**
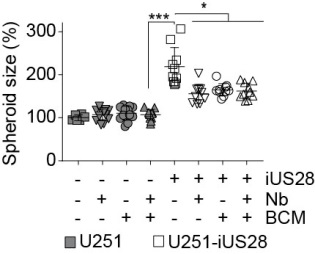
**

**Supplementary Figure 6** US28 nanobodies and BCM both inhibit US28-enhanced spheroid growth. Size of U251-iUS28 spheroids was determined upon treatment with US28-Nb, bevacizumab (BCM, 1.7 μM), or both in combination (n = 10 spheroids per group). *** P < 0.001, * P < 0.05 (unpaired t-test).

**
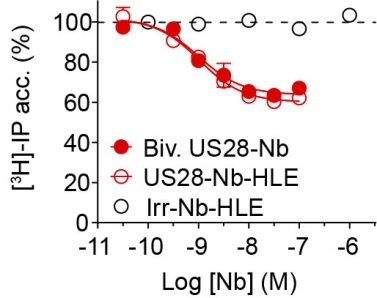
**

**Supplementary Figure 7** Half-life extended (HLE) nanobodies maintain their functionality towards US28. US28-mediated accumulation of ^3^H-labeled inositol phosphates (IP) in US28-expressing HEK239T cells upon treatment with concentration ranges of bivalent US28-Nb (closed red circles), US28-Nb^HLE^ (open red circles) or Irr-Nb^HLE^. Basal US28-mediated IP accumulation was set to 100% (dashed line).
